# The influence of home care supply on delayed discharges from hospital in England

**DOI:** 10.1186/s12913-021-07206-5

**Published:** 2021-12-02

**Authors:** Stephen Allan, Daniel Roland, Gintare Malisauskaite, Karen Jones, Kate Baxter, Kate Gridley, Yvonne Birks

**Affiliations:** 1 PSSRU, University of Kent, Canterbury, CT2 7NF UK; 2 Social Policy Research Unit, Department of Social Policy and Social Work, University of York, York, UK

**Keywords:** Delayed discharges, Delayed transfers of care, Home care, Health care, Social care, Supply

## Abstract

**Background:**

Delayed transfers of care (DTOC) of patients from hospital to alternative care settings are a longstanding problem in England and elsewhere, having negative implications for patient outcomes and costs to health and social care systems. In England, a large proportion of DTOC are attributed to a delay in receiving suitable home care. We estimated the relationship between home care supply and delayed discharges in England from 2011 to 2016.

**Methods:**

Reduced form fixed effects OLS models of annual DTOC attributed to social care at local authority (LA)-level from 2011 to 2016 were estimated, using both number of days and patients as the dependent variable. A count of home care providers at LA-level was utilised as the measure of home care supply. Demand (e.g. population, health, income) and alternative supply (e.g. care home places, local unemployment) measures were included as controls. Instrumental Variable (IV) methods were used to control for any simultaneity in the relationship between DTOC and home care supply. Models for DTOC attributed to NHS and awaiting a home care package were used to assess the adequacy of the main model.

**Results:**

We found that home care supply significantly reduced DTOC. Each extra provider per 10 sq. km. in the average local authority decreased DTOC by 14.9% (equivalent to 449 days per year), with a per provider estimate of 1.6% (48 days per year). We estimated cost savings to the public sector over the period of analysis from reduced DTOC due to increased home care provision between £73 m and £274 m (95% CI: £0.24 m to £545.3 m), with a per provider estimate of savings per year of £12,600 (95% CI: £900 to £24,500).

**Conclusion:**

DTOC are reduced in LAs with better supply of home care, and this reduces costs to the NHS. Further savings could be achieved through improved outcomes of people no longer delayed. Appropriate levels of social care supply are required to ensure efficiency in spending for the public sector overall.

## Introduction

Delayed transfers of care (DTOC) from hospital of patients medically fit to be discharged, colloquially and unfairly known as bed-blocking, is an important area for policy. In the UK, successive governments have made DTOC an area for NHS improvement through a number of mechanisms; introducing legislation, providing additional funds, e.g. Better Care Fund, and setting targets [1–3]. DTOC have become particularly important in recent years because of sustained increases over time [4].

Failure to discharge medically fit patients in a timely manner is generally thought to be the result of a combination of factors [1, 3]. Delays can occur within the hospital itself and can include the timing of ward rounds by doctors, who sign off a patient as being medically fit to be discharged, and the prescribing of medicines [3]. Poor discharge coordination, communication and planning can also influence unnecessarily long stays in hospital [1, 5]. Issues with funding or a lack of agreement over the discharge plan with family or the patient can further delay discharge [6].

DTOC will also be largely affected by the availability of social care [3, 6]. Social care is not part of the NHS; social care is the responsibility of local authorities (LAs) and funding for individuals is subject to eligibility criteria. Discharge from hospital may require both health and social care services, particularly for older frail patients, thus it is reliant on an adequate supply of appropriate social care, and home care in particular. Home care in England, also known as domiciliary care, refers specifically to short- or long-term services to support the social care needs of individuals living in their own homes, typically helping them with activities of daily living such as washing, dressing and cooking meals. Data for England shows that awaiting a care package to be provided in the patient’s own home was the highest cause of DTOC [4].

The vast majority of social care in England is now provided by independent providers - either private businesses or third sector organisations - and there has been increased emphasis within policy on prevention and receiving care in the community [7, 8]. In England, the market for home care has grown markedly over the last 20 years and there are now over 10,000 home care providers registered in England to provide these services. However, there is limited evidence on the effect that the increasing supply of home care is having on the health care system. Given this context, we looked to analyse the effect of home care supply on DTOC in England.

Internationally, there is limited systematic evidence as to what factors influence delayed discharges from hospital settings [9]. In England, research has found that care home bed supply reduces both DTOC and length of stay [10–13]. However, the effect of home care supply on DTOC is an under-researched area in England. One analysis utilised local authority (LA) funded home care usage data for England in 1998–2000, finding that greater support for discharge to home care weakly reduced emergency readmission rates but not DTOC, and that care home supply had a much larger effect [14]. Further research examining the impact on health care utilisation of LA expenditure on adult social care has found mixed results [15–17].

Social care in England is needs and means tested, and therefore demand for home care services comes from two main sources: private, self-funding individuals and from LAs that are (at least partially) paying for those that cannot (fully) afford to pay for their own care, although arrangements can be complex and change over time. The use of LA expenditure on home care, or on adult social care in general, as an indicator of social care supply (i.e. met demand) therefore suffers from a natural weakness in that it will not address the demand from private individuals paying for their own care. Nationally, it has been estimated that this market could be worth as much as £10.9bn a year, equivalent to about half the size of the public funded market [18].

We looked to build on the existing literature by utilising home care supply information from a national database of registered health and social care providers. We created measures of supply that treated the home care market both at the LA-level and by distance, i.e. across LA boundaries. Our working hypothesis was that higher availability of home care supply in a local market would reduce DTOC. The measurement of home care supply was not straightforward and we made a number of qualifying assumptions. Nonetheless, this is the first research to quantify the effects of home care supply on DTOC in England.

## Methods

### Data

The analysis used secondary data from 150 LAs, excluding Isles of Scilly and City of London. Annual data on DTOC at LA-level were collected from NHS England’s publically available ‘Delayed Transfers of Care Monthly Situation Report’ for 2011–2016 [19]. For the time period analysed, DTOC data included number of patients delayed, number of days delayed and the responsible organisation for the delay, the NHS, social care or both. This data was further broken down in to cause of delay.

### Social care supply

Data on home care and care home providers were taken from the Care Quality Commission’s (CQC) publically available ‘Care directory with filters’, a database of registered health and social care providers, at September of each year [20]. The CQC is the national health and social care regulator and all care homes are legally required to be registered with CQC to provide care services. Care home supply was measured as a count of beds in an LA. Whilst similar, for home care there are exemptions which mean not all providers are required to register [21]. For example, a self-employed carer who works directly for an individual who arranged and paid for their own care (as a private, self-funder or through a direct payment) would not need to register with the CQC. The analysis therefore only assessed the effect of registered home care providers on DTOC. We assumed that the register is a good proxy for overall home care levels in LAs. Given the large increase in registered providers observed in the data (see below), this did not seem unreasonable.

There were two further drawbacks to the data available from the CQC database. First, there was no available information on the size of home care providers. We therefore calculated home care supply in market size *y* as the total number of providers in the market, i.e. ${HC}_y=\frac{\sum_{j=1}^n{N}_j}{w^y}$, where *N* is the number of providers in the market and *w*^*y*^ is a specific weighting for each type of market size. In terms of a competition measure, if all home care providers in a market were of equal size then the count of providers would be the equivalent to the inverse of the Herfindahl-Hirschman Index (HHI), i.e. HHI = $N{\left(\overline{c_j}\right)}^2/{\left(N\overline{c_j}\right)}^2=1/N$, where *c* is the number of clients that each home care provider supports.

Second, there was no information of size of the market served. Home care provision will be delivered within the home and the size of the market served by providers will depend on local demand, competition and transport factors. Given the vast majority of providers of home care will serve a market close to their registered location, and as the best available information, the location (i.e. UK postcode) of where the provider was registered to provide care was used as an indication of location of market.

We measured market size *y* in two ways: by local authority (HC_LA_), and by distance (HC_10_). We weighted the former for size of LA, i.e. ${w}_i^{LA}={A}_i/10$, where *A* is the area of LA *i* in sq. km. HC_LA_ is therefore the number of providers per 10 sq. km. The latter was calculated in the following way. First, we counted the number of providers within 10 km of the centre of each Middle-layer Super Output Area (MSOA) in an LA. This was weighted by distance-weighted elderly population, i.e. ${w}_x^{10}={\sum}_{M=a}^z\left(\sqrt{d_a-{d}_z}\ast {e}_z\right)$, where *d* is the distance of MSOA *a* to *z* MSOAs that are within a 10 km radius and *e* is the per-1000 over-65 population in each of the *z* MSOAs. The total of all MSOAs was then averaged at LA-level, i.e. HC_10_ is the average number of providers per distance-weighted 1000 over-65 population within 10 km of each MSOA within an LA.

### Control variables

Data for other LA-level demand and supply characteristics that could influence DTOC and social care supply were drawn from Office for National Statistics, NHS Digital and the Land Registry, specifically: population of those aged 65 and over; number of hip fractures of those aged 65 and over; attendance allowance and pension credit uptake of those aged 65 and over as indicators of levels of needs and income, respectively; average house price as an indicator of wealth; gross adult social care expenditure per person as an indicator of demand for social care; and the percentage of those claiming job-seeker’s allowance as an indicator of the potential supply of informal carers. Finally, year fixed effects were also included.

### Analysis

We estimated the following reduced form model of LA-level DTOC:

1 $$ {DTOC}_{it}={\alpha}_i+{\gamma}_t+{\beta S}_{it}+{\sigma X}_{\boldsymbol{it}}+{u}_{it} $$

**Fig. 1:**
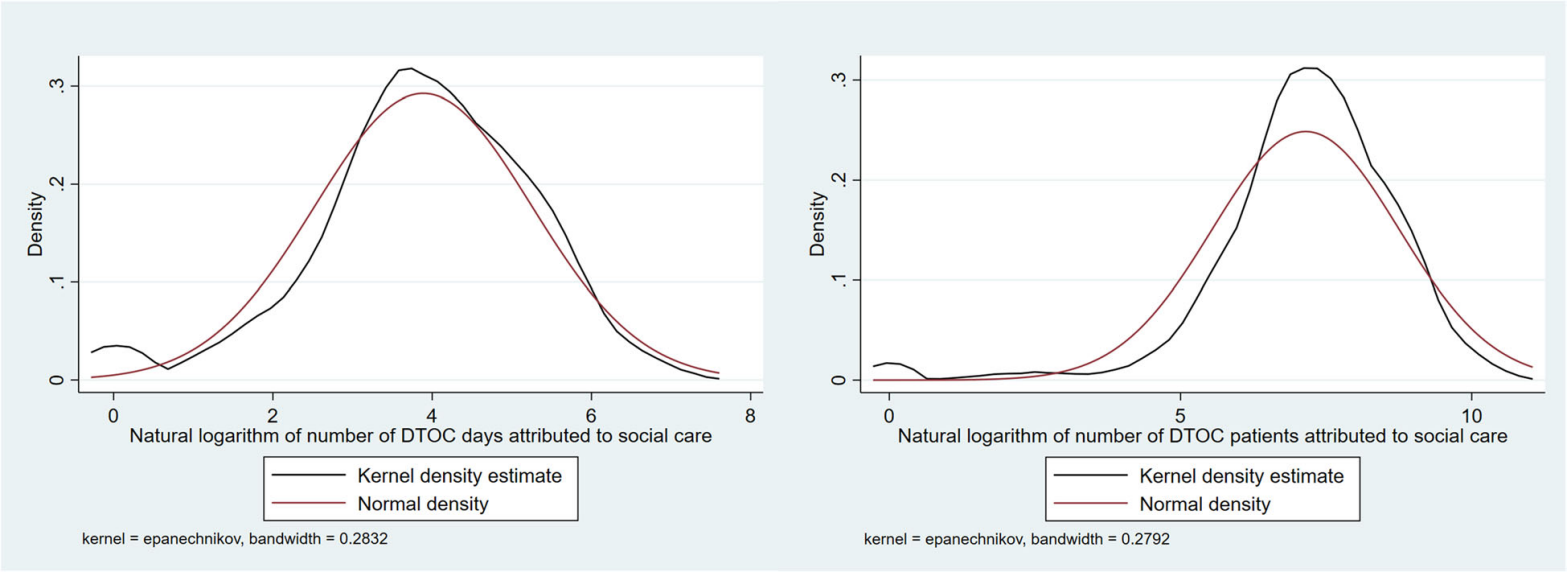
Kernel density plots of natural logarithm of DTOC attributed to social care, days and patients

Where *DTOC*_*it*_ is (log) total number of patient days or number of patients delayed in hospitals in LA *i* in year *t*, *α* is an LA-effect, *γ* is a year effect, *S* is the measure of home care supply, *X* is a vector of demand, needs and supply measures, including care home beds, and *u* is the residual error term. The dependent variable was DTOC where social care was detailed as the responsible organisation. We estimated the model of DTOC due to social care using OLS, employing fixed effects specifications, the choice of which was determined from Mundlak and Hausman tests [22, 23], and with robust standard errors clustered at LA-level. This followed previous research [10] and we found the natural logarithm of both DTOC days and patients to be approximately normally distributed (see Fig. 1).

It is plausible that DTOC and home care supply could be jointly determined. For example, areas with high DTOC may have increased home care supply to help mitigate a lack of available hospital beds. This positive relationship would be opposite to the expected relationship, reducing the expected true effect of home care supply on DTOC. As such, to control for possible simultaneity, we used the 1 year lag of home care supply as an instrument in instrumental variables (IV) model specifications.

To be an appropriate instrument, the time lag of home care supply is required to be correlated with current home care supply but not correlated with *u*_*it*_. The latter could occur, for example, if home care supply feeds in to current DTOC levels through past DTOC levels causing backlogs in patients leaving hospital. However, over the period analysed, average length of stay in English hospitals was around 5 days [24]. A potentially more likely avenue for the instrument being spuriously correlated with the error term is because of non-parallel trends, e.g. the needs of populations changing over time to different extents in markets with different levels of home care supply.

We assessed the adequacy of the instrument in the following ways. First, the strength of the relationship between current and past social care supply was assessed using a robust Wald test of past supply in a (first stage) regression of current home care supply. To assess for correlation between the instrument and the model error term, we descriptively examined the difference in DTOC between LAs with high and low levels of home care supply across the period analysed. Further, to control for potential non-parallel trends in the regression models, we assessed if results changed when including interactions between the controls and year fixed effects. Ultimately, we assessed strict exogeneity of the instrument using a variable addition test, i.e. we included the 1-year forward lag of home care supply in models and assessed if it significantly influenced DTOC [25]. A significant test finding would suggest that the instrument is correlated with *u*_*it*_ and the model is inconsistent.

We assessed the validity of our findings using delays attributed to NHS and also for delays attributed to the cause of waiting for a home care package. The first of these was used as a placebo test, i.e. if there was a significant effect on NHS delays then this would give an indication of potential omitted variable bias. The latter data was used to analyse if the effect is stronger on delays attributed to awaiting home care packages specifically and could be considered an upper bound on size of effect. We also estimated an unweighted per provider effect by including the count of home care providers at LA-level in the main analysis (HC).

Finally, we estimated cost savings to the public purse from reduced DTOC due to increased home care. Using National Audit Office (NAO) figures of £303 per day of DTOC and £41 per day of home care [26] and assuming all LAs are equal, we estimated per year savings from reduced DTOC days due to increased home care supply compared to 2011.

## Results

Table 1 reports the number of registered home care providers by region of England for 2011–2016. The number of providers registered increased by over 54% in the 5 year period and in October 2016 there were almost 7000 registered providers of home care for older people.

**Table 1:** Home care providers for older people, by year

Region	2011	2012	2013	2014	2015	2016
East Midlands	360	447	496	550	596	669
East of England	475	590	659	704	760	810
London	592	772	831	878	933	987
North East	198	222	241	249	254	252
North West	564	714	762	813	851	847
South East	825	1001	1069	1084	1117	1168
South West	511	603	649	673	701	719
West Midlands	585	678	718	765	828	898
Yorkshire & Humber	413	494	548	594	614	620
England	**4523**	**5521**	**5973**	**6310**	**6654**	**6970**

*Notes*: Social care organisation (i.e. non-health) home care providers registered to provide care for older people and/or those living with dementia. Source: CQC database of registered health and social care providers. Excludes City of London and Isles of Scilly

Tables 2 and 3 provide information at LA-level on social care supply over time and the variables included in the analysis, respectively. Table 2 shows that the average LA had 16 more providers of home care in 2016 compared to 2011, and this is reflected in the measures of home care supply HC_LA_ and HC_10_. The number of providers per 10 square km increased by more than one from 2.06 providers in 2011 to 3.21 in 2016, and each MSOA in the average LA had half a provider extra per 1000 elderly population within a 10 km radius in 2016 compared to 2011. Care home beds showed a modest increase over the period analysed. Table 3 generally shows large variations by LA in DTOC by both days and patients and for social care supply. The average LA had over 3000 days of DTOC and 105 patients delayed per year attributed to social care, 2700 care home beds and 2.75 home care providers per 10 sq. km.

**Table 2:** Local authority-level social care supply measures (mean), by year

Measure	2011	2012	2013	2014	2015	2016
Total HC providers (HC)	30.15	36.81	39.82	42.07	44.36	46.47
HC providers per 10 sq. km (HC_LA_)	2.06	2.54	2.73	2.89	3.06	3.21
HC providers within 10 km of each MSOA in LA per 1000 65+ pop. (HC_10_)	1.22	1.46	1.54	1.59	1.66	1.72
Care home beds	2633.1	2701.6	2715.83	2722.9	2721.2	2711.02

*Notes*: *HC* Home Care, *pop* population

**Table 3:** Descriptive statistics

Variable	mean	S.D.	Min	25th pc	median	75th pc	max
**Delayed Transfers of Care**							
Days (Social care)	3017.08	4475.98	0	668.5	1464	3406.5	47,452
Patients (Social care)	105.51	148.89	0	24	52	126.5	1515
Days (NHS)	6521.96	7189.13	25	2100.5	3661.5	7538.5	48,844
Patients (NHS)	226.57	241.82	3	75	130	275	1645
Days (AHCP)	1426.28	2575.39	0	154	496.5	1485.5	25,759
Patients (AHCP)	49.61	84.29	0	6	19	54.5	829
**Home care supply**							
HC_LA_	2.75	2.96	0.04	0.48	1.46	4.53	16.69
HC_10_	1.53	0.50	0.53	1.17	1.43	1.85	3.67
**Control variables**							
Care home beds	2701	2524	246	1119	1753	3323	12,847
JSA claimants (%)	2.85	1.62	0.39	1.59	2.54	3.88	8.76
ASC exp. per pop 16+ (£)	0.318	0.077	0.139	0.255	0.313	0.374	0.623
Population 65+	62,459	55,516	7962	28,481	38,977	70,607	305,924
Avg. house price (£)	264,722	203,669	97,165	152,265	210,327	296,036	1,947,723
PC uptake (%)	24.12	9.55	6.85	17.11	22.51	29.29	68.09
AA uptake (%)	13.59	2.57	7.63	11.83	13.23	15.15	24.04
Hip fractures 65+	377.87	338.21	0	165.5	246.5	426	1882

*Notes*: *n* = 900, *AHCP* Awaiting home care package, *JSA* Job Seeker’s Allowance, *ASC exp. per pop 16+* Adult Social Care expenditure per population over 16 years of age, *PC* Pension Credit, *AA* Attendance Allowance

**Fig. 2:**
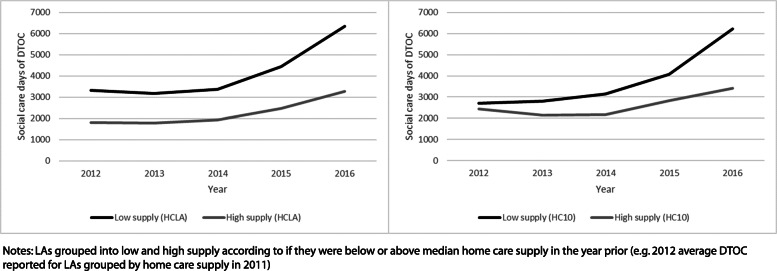
Average LA social care days of DTOC, by home care supply instrument (low vs high)

Figure 2 shows the number of days of DTOC attributable to social care for LAs by year when split in two groups according to their level of home care supply in the preceding year (i.e. the instrument), measured by HC_LA_ and HC_10_, respectively. DTOC rates on average are higher in LAs that are in the lower half of the home care supply distribution across the period analysed. There is limited evidence of a divergence in levels of DTOC between the two groups of LAs across time for HC_LA_, with the largest difference occurring in 2016. However, there is a greater suggestion of divergence when LAs are grouped by HC_10_.

Tables 4 and 5 reports the results for the estimation of eq. 1, which models delayed discharges in England due to social care when measured by total days (first two columns) and number of patients delayed (latter two). The odd columns in both tables report the basic exogenous home care supply model and the even columns report the IV specifications. Table 4 reports results when including HC_LA_ as the measure of home care supply and Table 5 results when including HC_10_.

**Table 4:** Results of models of delayed discharges attributed to social care with HC_LA_

	Log DTOC (days)	Log DTOC (days)	Log DTOC (patients)	Log DTOC (patients)
	FE	IV FE	FE	IV FE
HC_LA_	−0.140^c^	− 0.149^b^	− 0.150^c^	−0.184^b^
	(0.0359)	(0.0756)	(0.0380)	(0.0774)
Care home beds (log)	−0.731	− 0.799	−1.188^a^	−1.320^a^
	(0.638)	(0.706)	(0.700)	(0.773)
JSA claimants (%)	−0.166^a^	−0.134	− 0.227^a^	−0.175
	(0.0997)	(0.128)	(0.119)	(0.146)
ASC exp. per pop 16+	2.375^b^	1.961^a^	3.396^b^	3.059^b^
	(1.097)	(1.093)	(1.350)	(1.394)
Population 65+ (log)	−0.691	−3.157	1.606	−1.641
	(2.529)	(3.056)	(2.796)	(3.335)
Avg. house price (log)	−0.0151	− 0.347	−0.171	− 0.677
	(0.671)	(0.772)	(0.813)	(0.901)
PC uptake (%)	0.0894^a^	0.0907	0.1004^b^	0.09629
	(0.0460)	(0.0692)	(0.0499)	(0.0714)
AA uptake (%)	0.1050^a^	0.1410^a^	0.1213	0.1984^b^
	(0.0631)	(0.0723)	(0.0741)	(0.0914)
Hip fractures 65+	0.109	0.0423	0.181^b^	0.0911
	(0.0657)	(0.0597)	(0.0698)	(0.0613)
Years (*γ*)	YES	YES	YES	YES
N	900	750	900	750
R^2^	0.269	0.263	0.244	0.234
Hausman test	35.48^c^	35.48^c^	35.45^c^	35.45^c^
Mundlak test	28.78^c^	28.78^c^	27.41^c^	27.41^c^
Weak instruments (HC_LA_)		25.71^c^		25.71^c^
Strict exogeneity (HC_LA_)		−0.48^NS^		−0.81^NS^

*Notes*: *FE* Fixed Effects, *IV* Instrumental Variables, *DTOC* Delayed Transfers of Care, *JSA* Job Seeker’s Allowance, *ASC exp. per pop 16+* Adult Social Care expenditure per population over 16 years of age, *PC* Pension Credit, *AA* Attendance Allowance, *NS* Not Significant. Standard errors in parentheses. ^a^, ^b^, and ^c^ indicate significance at 10, 5 and 1%, respectively

**Table 5:** Results of models of delayed discharges attributed to social care with HC_10_

	Log DTOC (days)	Log DTOC (days)	Log DTOC (patients)	Log DTOC (patients)
	FE	IV FE	FE	IV FE
HC_10_	−0.562^c^	− 1.057^b^	− 0.587^c^	−1.127^b^
	(0.185)	(0.415)	(0.210)	(0.502)
CH beds per 1000 65+	−0.0249	−0.0182	− 0.0412^b^	−0.0360
	(0.0174)	(0.0221)	(0.0189)	(0.0250)
JSA claimants (%)	−0.177^a^	−0.215^a^	− 0.201^a^	−0.228^a^
	(0.0951)	(0.121)	(0.110)	(0.138)
ASC exp. per pop 16+	2.175^b^	1.791^a^	3.239^b^	2.832^b^
	(1.059)	(1.069)	(1.306)	(1.364)
Avg. house price (log)	−0.0123	0.0776	−0.133	− 0.275
	(0.666)	(0.786)	(0.828)	(0.921)
PC uptake (%)	0.1149^b^	0.1372^b^	0.1240^b^	0.1415^b^
	(0.0462)	(0.0637)	(0.0506)	(0.0672)
AA uptake (%)	0.0975	0.1113	0.1172	0.1694^a^
	(0.0640)	(0.0764)	(0.0734)	(0.0955)
Hip fractures per 1000 65+	0.0261	−0.0122	0.0478	−0.00740
	(0.0405)	(0.0428)	(0.0458)	(0.0473)
Years (*γ*)	YES	YES	YES	YES
N	900	750	900	750
R^2^	0.269	0.262	0.244	0.235
Hausman test	32.79^c^	32.79^c^	31.41^c^	31.41^c^
Mundlak test	21.62^b^	21.62^b^	19.61^b^	19.61^b^
Weak instruments (HC_10_)		26.96^c^		26.96^c^
Strict exogeneity (HC_10_)		0.98^NS^		1.05^NS^

*Notes*: *FE* Fixed Effects, *IV* Instrumental Variables, *DTOC* Delayed Transfers of Care, *CH* Care Home, *JSA* Job Seeker’s Allowance, *ASC exp. per pop 16+* Adult Social Care expenditure per population over 16 years of age, *PC* Pension Credit, *AA* Attendance Allowance, *NS* Not Significant. Standard errors in parentheses. ^a^, ^b^, and ^c^ indicate significance at 10, 5 and 1%, respectively

Both measures of home care supply are significant and negative in their influence on DTOC in all specifications for both days and patients delayed. The size of effect of home care supply on DTOC varied depending on the supply measure utilised and the form of DTOC. Using IV models increased the (absolute) size of the coefficient, suggesting a positive bias from treating the relationship between DTOC and home care supply as exogenous. From the IV models, an increase of one provider per 10 sq. km. in an LA reduced DTOC days (patients) attributed to social care by 14.9% (95% Confidence Interval [CI]: 0.05 to 29.7%), which is equivalent to 449 days per year for the average LA in the sample. The equivalent figure for DTOC patients were 18.4% (95% CI 3.2 to 33.5%) and 19 patients per year. Increasing by one the average number of providers per 1000 over-65 population within 10 km of each MSOA within an LA reduced social care DTOC days (patients) by 106% (95% CI: 24.3 to 186.7%), or 3198 days per year for the average LA. For DTOC patients, the same figures were 113% (95% CI: 14.3 to 211.1%) and 118 patients per year.

As shown in Tables 4 and 5, the 1 year lag of HC_LA_ and HC_10_ were strong instruments (weak instruments test) and there was no indication in the IV models of unidentified correlation between each instrument and DTOC (strict exogeneity test). When estimating the IV models of DTOC from Tables 4 and 5 including interactions of the controls with year fixed effects, we found little evidence of a reduction in size of effect of home care supply, and only in one of the four models (Days, HC_LA_) was the significance level below ρ < 0.05. Overall, we concluded that it was unlikely that the instruments of home care supply were correlated with DTOC.

We did not find consistent evidence of a significant negative care home effect on DTOC and the size of effect varied. We believed this to be caused from there being little change in bed supply from year to year within LAs. For example, utilising a random effects specification, we found a similar effect to that of [10], with a 10% rise in care home beds significantly reducing social care DTOC days by 7.6%.

### NHS DTOC

Table 6 reports the marginal effects of the two home care supply measures from estimating further models of DTOC as extensions to the main analysis. The first two rows of Table 6 report the marginal effects of the supply measures when considering a model of DTOC attributable to the NHS. The size of relationship between NHS DTOC and home care supply are smaller and only significant in specifications using the HC_LA_ measure of home care supply when assuming exogeneity between HC_LA_ and DTOC.

**Table 6:** Marginal effects of home care supply on various DTOC

	Log DTOC (days)	Log DTOC (days)	Log DTOC (patients)	Log DTOC (patients)
**Type of DTOC/Home care measure**	**FE**	**IV FE**	**FE**	**IV FE**
NHS: HC_LA_	−0.0469^c^	− 0.0413	− 0.0416^b^	−0.0459
	(0.0173)	(0.0408)	(0.0196)	(0.0400)
NHS: HC_10_	0.0182	−0.0045	− 0.0363	−0.0903
	(0.1315)	(0.3020)	(0.1335)	(0.2861)
AHCP: HC_LA_	−0.143^c^	−0.210^b^	− 0.0944^a^	−0.256^a^
	(0.051)	(0.103)	(0.0501)	(0.136)
AHCP: HC_10_	−0.872^c^	− 1.234^b^	− 0.541^c^	− 1.009^b^
	(0.266)	(0.626)	(0.201)	(0.492)
Social care: HC	−0.0110^b^	− 0.0159^b^	− 0.0133^b^	− 0.0206^b^
	(0.0048)	(0.0076)	(0.0056)	(0.0094)
Weak instruments (HC_LA_) NHS		25.71^c^		25.71^c^
Weak instruments (HC_LA_) ACHP		25.71^c^		9.63^c^
Weak instruments (HC_10_) NHS		26.96^c^		26.96^c^
Weak Instruments (HC_10_) AHCP		15.74^c^		15.74^c^
Weak Instruments (HC) SC		100.93^c^		100.93^c^
Strict exogeneity (HC_LA_) NHS		−1.73^a^		0.64^NS^
Strict exogeneity (HC_10_) NHS		−0.83^NS^		−0.20^NS^
Strict exogeneity (HC_LA_) AHCP		−0.52^NS^		−0.97^NS^
Strict exogeneity (HC_10_) AHCP		1.92^b^		1.45^b^
Strict exogeneity (HC) SC		0.46^NS^		0.75^NS^

*Notes*: *FE* Fixed Effects, *IV* Instrumental Variables, *DTOC* Delayed Transfers of Care, *AHCP* Awaiting Home Care Package, *SC* Social Care, *NS* Not Significant. All models include control variables from Tables 4 and 5, respectively. AHCP models use two lags of respective home care supply measure as instruments for DTOC days (HC_10_ only) and DTOC patients. Standard errors in parentheses. ^a^, ^b^, and ^c^ indicate significance at 10, 5 and 1%, respectively

### Awaiting home care package DTOC

The third and fourth rows of Table 6 report the marginal effects of the respective supply measures from estimating models of DTOC where the cause for delay was awaiting a home care package. The size of effect for the measures of home care supply were larger for all IV specifications of the awaiting home care package DTOC model with the exception of HC_10_ and DTOC patients. These can be considered an upper bound on size of effect. For example, each extra provider per 10 sq. km in an LA reduced days of DTOC by 21.0% (95% CI: 0.8 to 41.2%). However, we note that strict exogeneity was rejected for HC_10_ in both DTOC days and patients models.

### Per provider effect

The final row of Table 6 reports the marginal effects from estimating the model of social care DTOC for days and patients when including the unweighted count of providers at LA-level (HC) in the analysis. The IV results suggest that for the average LA each extra provider would reduce social care DTOC days by 1.6% (95% CI: 0.11 to 3.1%) and patients by 2.1% (95% CI: 0.21 to 3.9%). This is equivalent to 48 days per year or 2 patients per year for the average LA.

### Cost savings

Table 7 reports the estimated cost savings from increasing home care. For example, in 2016 the average LA had an increase compared to 2011 of 1.15 providers per 10 sq. km and 0.5 providers per 1000 elderly population within 10 km of each MSOA. These suggest savings in 2016 compared to 2011 of £20.3 m (HC_10_) or £62.8 m (HC_LA_). In total, savings were estimated at £72.9 m (HC_LA_) or £235.0 m (HC_10_) for England over the period analysed. For comparison, Table 7 also presents the same findings for awaiting a home care package DTOC, which provide higher estimated total savings over the period of analysis by £29.8 m (HC_LA_) and £38.6 m (HC_10_). The table also shows, however, the wide confidence range around these figures.

**Table 7:** Estimated national cost savings from reduced DTOC due to increased home care supply

Type of DTOC/Home care measure	2012	2013	2014	2015	2016	Total	Total 95% CI
Social Care: HC_LA_	£8.5 m	£11.8 m	£14.6 m	£17.6 m	£20.3 m	**£72.9 m**	**£0.24 m-£145.4 m**
Social Care: HC_10_	£30.2 m	£40.2 m	£46.5 m	£55.3 m	£62.8 m	**£235.0 m**	**£53.9 m-£414.5 m**
AHCP: HC_LA_	£11.9 m	£16.7 m	£20.6 m	£24.9 m	£28.6 m	**£102.7 m**	**£3.7 m-£201.8 m**
AHCP: HC_10_	£35.1 m	£46.8 m	£54.1 m	£64.4 m	£73.2 m	**£273.6 m**	**£1.5 m-£545.3 m**
Social Care: HC	£12.6 m	£18.2 m	£22.5 m	£26.8 m	£30.8 m	**£110.8 m**	**£6.4 m-£216.0 m**

*Notes*: Savings in comparison to 2011 home care supply. *CI* Confidence interval, *AHCP* Awaiting home care package. Total may not be equal to the sum of years due to rounding

The HC measure of supply allowed for an estimate of per year savings from an extra home care provider. Each extra provider in the average LA would create savings of £12,600 per year (95% CI: £900 to £24,500) and, using this figure, overall savings in the time period analysed are estimated to have been £110.8 m nationally (Table 7). For comparison, we also estimated per provider savings from the estimated national savings per year for HC_LA_ and HC_10_, finding per provider savings to the health and social care system of £8200–8500 per year (HC_LA_) or £25,700–£30,200 per year (HC_10_). The same figures from the awaiting home care package DTOC models were £11,500–£12,000 per year (HC_LA_) and £29,900–£35,200 per year (HC_10_).

## Discussion

DTOC are an ongoing issue in the English health and social care system and reducing DTOC has been an important target in NHS policy. A large number of DTOC are attributed to a lack of available home care. This research has analysed the association of home care supply on DTOC using data at the local authority level for the years 2011–2016, measuring home care supply of CQC registered providers, and controlling for need, demand and supply characteristics, including care home supply. The findings suggest that DTOC and home care supply have a significant inverse relationship. Every extra home care provider per 10 sq. km. within an LA reduced the number of days delayed due to social care by almost 15%, or the equivalent of 449 days per year for the average LA.

DTOC have wide-ranging implications for the health and social care system [27, 28]. These include the cost implications for any delayed transfer [29]. In England, it has been estimated that DTOC cost the NHS over £820 m a year [26]. Whilst there would be additional costs to health and social care from supporting delayed patients in settings other than the NHS, these are likely to be appreciably lower, and particularly in an individual’s own home. The results in our analysis confirmed this, suggesting direct savings of £12,600 per year from reduced DTOC through the addition of one extra home care provider in the average LA. Although these savings appear modest, they could have important cost implications to the health and social care system overall. Our estimates suggest that over the period 2011–2016 the increase in home care supply may have provided savings to the public sector in the range £73 m to £274 m, although some caution must be taken when interpreting these figures. Nonetheless, these savings would be an underestimate as a) some proportion of the home care purchased would be self-funded by private payers and b) the ongoing costs to health and social care system from health deterioration due to DTOC are unknown (see below). Ultimately, it is important that there is an appropriate level of social care supply available to achieve potential savings across the health and social care system [30].

Further, DTOC and the availability of social care could impact on user outcomes. A number of negative outcome indicators have been associated with delayed hospital discharge which include frailty, cognitive impairment and reduced ability to undertake activities of daily living [29, 31–35]. As such, it is important that the appropriate level of social care supply is available to support those who are leaving hospital and returning to the community, enhancing quality of life and preventing admissions and readmissions [36]. Research would be required to establish if further cost savings could be made to the public sector from reducing any potential greater future need for health and social care resulting from DTOC.

Previous analyses have examined in detail the relationship between healthcare utilisation and LA adult social care expenditure as an indicator of home care supply [15–17]. In this analysis, we controlled for adult social care expenditure in order to assess a true supply effect, i.e. one not driven by LAs’ spending on social care. Therefore, importantly, this study adds to the health and social care substitution literature by finding that utilisation of healthcare depends not just on adult social care expenditure, although noting the mixed findings in this regard, but also on available home care supply. This is consistent with previous literature for England, particularly when looking at care homes [10, 14]. Ultimately, the results of this paper support and extend the finding that length of stay in hospitals was significantly lower in LAs with the highest levels of independent sector social care staffing [17].

### Limitations

The analysis depends on the quality of the data, including the level of accuracy in the reporting of DTOC. There are also limitations to the measures of home care supply employed. First, the size of each provider is unknown. Second, whilst the market size utilised reflects where social care commissioning decisions are made, i.e. LA-level, each of the supply measures we used do not allow for variation in size of markets, e.g. across LA borders, only certain areas of cities etc. Information on price or quality of home care supply were not available for the analysis, which may impact on the findings. Cost savings estimates have a wide range of confidence but are likely to be an underestimate, as noted above. Heterogeneous variation in DTOC and home care supply will also impact on these savings estimates. Future research would require more granular data to estimate market size and utilise any data on service users that become available to confirm and refine the results found in this analysis.

## Conclusion

DTOC are significantly reduced with an increased number of home care providers in a local market, and this could have important cost saving implications for the NHS. Further cost savings could be achieved through improved outcomes of patients no longer delayed in hospital. Therefore, appropriate levels of funding and support to develop the social care market could increase efficiency in spending for the health and social care sectors overall. This analysis provides evidence that health and social care systems are inexorably linked and that to deliver effective and efficient health care the supply of social care for the older community-dwelling population should also be considered.

## Data Availability

The dataset generated during and analysed during the current study are available from the corresponding author on reasonable request.
